# Beneficial Metabolic Effects of CB1R Anti-Sense Oligonucleotide Treatment in Diet-Induced Obese AKR/J Mice

**DOI:** 10.1371/journal.pone.0042134

**Published:** 2012-08-03

**Authors:** Yuting Tang, George Ho, Yaxin Li, Meghan A. Hall, Robert L. Hills, Shawn C. Black, Yin Liang, Keith T. Demarest

**Affiliations:** Cardiovascular and Metabolism Therapeutic Area, Janssen Pharmaceutical Companies of Johnson and Johnson, Spring House, Pennsylvania, United States of America; Hosptial Infantil Universitario Niño Jesús, Spain

## Abstract

An increasing amount of evidence supports pleiotropic metabolic roles of the cannibinoid-1 receptor (CB1R) in peripheral tissues such as adipose, liver, skeletal muscle and pancreas. To further understand the metabolic consequences of specific blockade of CB1R function in peripheral tissues, we performed a 10-week-study with an anti-sense oligonucleotide directed against the CB1R in diet-induced obese (DIO) AKR/J mice. DIO AKR/J mice were treated with CB1R ASO Isis-414930 (6.25, 12.5 and 25 mg/kg/week) or control ASO Isis-141923 (25 mg/kg/week) via intraperitoneal injection for 10 weeks. At the end of the treatment, CB1R mRNA from the 25 mg/kg/week CB1R ASO group in the epididymal fat and kidney was decreased by 81% and 63%, respectively. Body weight gain was decreased in a dose-dependent fashion, significantly different in the 25 mg/kg/week CB1R ASO group (46.1±1.0 g vs veh, 51.2±0.9 g, p<0.05). Body fat mass was reduced in parallel with attenuated body weight gain. CB1R ASO treatment led to decreased fed glucose level (at week 8, 25 mg/kg/week group, 145±4 mg/dL vs veh, 195±10 mg/dL, p<0.05). Moreover, CB1R ASO treatment dose-dependently improved glucose excursion during an oral glucose tolerance test, whereas control ASO exerted no effect. Liver steatosis was also decreased upon CB1R ASO treatment. At the end of the study, plasma insulin and leptin levels were significantly reduced by 25 mg/kg/week CB1R ASO treatment. SREBP1 mRNA expression was decreased in both epididymal fat and liver. G6PC and fatty acid translocase/CD36 mRNA levels were also reduced in the liver. In summary, CB1R ASO treatment in DIO AKR/J mice led to improved insulin sensitivity and glucose homeostasis. The beneficial effects of CB1R ASO treatment strongly support the notion that selective inhibition of the peripheral CB1R, without blockade of central CB1R, may serve as an effective approach for treating type II diabetes, obesity and the metabolic syndrome.

## Introduction

It has been well established that the endocannabinoid system consisting of CB1R and CB2R and their endogenous ligands (anandamide and 2-arachidonoylglycerol) play a significant role in regulating multiple metabolic pathways [1], [2], [3]. Initially, it was believed that CB1 receptor was predominantly localized in the central nervous system, while CB2 receptor was mainly expressed in peripheral cells and tissues from the immune system. Recently, CB1 receptors were also found in peripheral tissues such as adipose, liver, gastrointestinal tract (e.g., vagal afferent neurons, ileum longitudinal smooth muscle), skeletal muscle, and pancreas [4], [5], [6], [7], [8], [9]. Activation of CB1 receptors triggers many physiological processes, both centrally and peripherally [10], [11], [12]. CB1 receptors in the hypothalamus play a key role in food intake and energy homeostasis [13], [14]. Early work by Di Marzo et al demonstrated that defective leptin signaling pathway was associated with elevated endocannibinoids level in the hypothalamus which in turn over-stimulated CB1 receptors and increased food intake [14]. Moreover, overactivation of the endocannabinoid system in peripheral tissues such as adipose, pancreas and liver has been linked to obesity and the metabolic syndrome in both obese animals [15], [16] and humans [15], [17], [18], [19]. In recent years, emerging evidence has supported the notion that blockade of CB1 receptors with antagonists in peripheral tissues may provide sufficient metabolic benefits in feeding through gut-brain signaling [20], [21], [22], adipose tissue metabolism [23], [24], hepatic lipogenesis [23], glucose homeostasis, insulin release in the pancreas [8], [25], [26], cholesterol metabolism in macrophages [27] and metabolic control in skeletal muscle [28]. Since CB1 receptors are detected in many other central nervous regions influencing key functions, such as mood, motor coordination, and cognition [29], [30], administration of centrally penetrant CB1 receptor antagonists such as rimonabant has been associated with psychiatric risks [10], [11]. Therefore, targeting CB1 receptors in peripheral tissues has emerged to be a promising therapeutic approach to treat obesity, diabetes and the metabolic syndrome (for review, see [31]). To this end, we utilized the anti-sense oligonucleotide approach to evaluate the metabolic effects upon blockade of peripheral CB1R in diet-induced obesity AKR/J mouse model.

## Methods

### CB1R ASO and ASO Control

CB1R-ASO used in this study was Isis-414930; scrambled control ASO was Isis-141923. To identify mouse CB1R ASO inhibitors, rapid throughput screens were performed in vitro and several potent and specific ASOs were identified, all of which targeted a binding site within the coding region of the CB1R. After extensive dose response characterization, the most potent ASO from the screen was chosen: ISIS-414930, with the following sequence: 5- AGGTAGCTTAACGCACACAT -3. The control ASO, ISIS-141923, has the following sequence, 5 -CCTTCCCTGAAGGTTCCTCC-3, and does not have perfect complementarily to any known gene in public data bases. All ASOs were made in saline at appropriate concentrations.

### 10-week Study with DIO Male AKR/J Mice

All of the procedures for animal studies were approved by the Janssen Pharmaceutical Companies Institutional Animal Care and Use Committee. Food and water were supplied ad libitum. Room temperature was maintained at 68–72 F and humidity at 50–65%. Room lighting was on a 12-h light/12-h dark cycle. Male AKR/J mice from the Jackson Lab were single-housed and fed D12451 (45% high fat, Research Diets, New Brunswick, NJ) at 7–8-week old. At the initiation of study, mice were on D12451 for 10-week and at the age of 17–18-week. Age-matched lean mice were fed with standard rodent diet (LabDiet 5001, PMI Nutrition Int’l, St. Louis, MO). Mice (n = 10 per group) were treated subcutaneously with CB1R ASO (Isis-414930) and control ASO Isis-141923 dissolved in saline twice a week via i.p. injection for 10 weeks. For CB1R ASO, the mice were treated at 6.25, 12.5 or 25 mg/kg/week. Such dosing scheme provides sustained coverage based on our experience with these ASOs (data not shown). For scrambled control ASO, the mice were treated at 25 mg/kg/week. Body weight and food intake was monitored weekly. Fed blood glucose was obtained in the postprandial state at 9∶00–10∶00 AM. Body composition was assessed by mouse whole body Bruker magnetic resonance analyzer (Woodlands, TX). Oral glucose tolerance test (OGTT) was performed during week 9. For OGTT, all mice were fasted overnight and orally dosed with 2 g/kg glucose (20% glucose at 10 mL/kg). Blood glucose was measured at 0, 30, 60, 120 min after glucose dosing. Necropsy was performed during week 10. The mice were treated with the last dose 18-hour prior to the necropsy. Food was removed for 4-hour prior to the necropsy. Retro-orbital bleeding after 70% CO2/30% O2 anesthesia was performed to collect plasma for determination of triglycerides, total cholesterol, adiponectin, insulin, and leptin. Liver, spleen, epididymal fat, and kidney were dissected for total weight and/or gene expression analysis. Plasma glucose, triglycerides and total cholesterol levels were analyzed on Cobas Mira clinical chemistry analyzer (Roche Molecular Diagnostics, Pleasanton, CA). Clinical chemistry parameters such as creatinine, blood urea nitrogen (BUN), lipase, alanine aminotransferase (ALT), aspartate aminotransferase (AST) were also measured on Cobas Mira clinical chemistry analyzer. Total adiponectin was measured using mouse total adiponectin Elisa kit (Alpco Diagnostics, Salem, NH). Leptin was measured using mouse leptin ELISA kit Insulin was measured with HTRF insulin assay (Cis-bio, Bedford, MA). Mouse cytokine kit MADPK-71 K from Millipore was used to measure plasma total plasminogen activator inhibitor 1 (PAI-1), TNF alpha and IL-6 levels on Luminex 200™ (Millipore, Billerica, MA). For liver TG determination, liver was homogenized and lipid was extracted. Liver TG was then determined with WAKO L-type TG-M assay (WAKO, Richmond, VA) and normalized with tissue weight.

### Real-Time Quantitative PCR Analysis of Gene Expression

RNA was extracted from tissues using Qiazol lysis buffer according to the manufacturer’s instructions (Qiagen, Valencia, CA). Real-time quantitative PCR reactions were carried out using the TaqMan Gene Expression Assay Universal PCR Master Mix and an ABI Prism 7900 Sequence Detection System (Applied Biosystems, Foster City, CA). Comparative C_T_ method (ΔΔC_T_ method) was used following the instructions of the manufacturer (Applied Biosystems). Briefly, average C_T_ value from three reactions for a particular gene mRNA of each animal was normalized to the level of its own beta actin to generate ΔC_T_, followed by ΔΔC_T_ calculation (ΔΔC_T_ = ΔC_T_-average ΔC_T_ of vehicle group). Fold change of a particular gene mRNA of each animal from that of vehicle group was then obtained by calculating 2^−ΔΔC^_T_. Subsequently, an average of fold change and standard error were derived. All primers were purchased from Applied Biosystems (Mm00432621_s1 for CB1R, Mm00438286_m1 for CB2R, Mm01138344_m1 for SREBP1, Mm00839363_m1 for G6PC, Mm00432403_m1 for CD36).

### Statistical Analysis

Statistic analysis was performed using Graphpad Prism (Monrovia, CA, USA) with either one-way ANOVA Dunnett’s multiple comparison test or student t-test two-tailed distribution homoscedastic analysis.

## Results

### CB1R ASO Treatment Decreased CB1R mRNA Levels in WAT and Kidney of DIO AKR/J Mice

In the current study, we treated DIO AKR/J mice with a specific and potent CB1R ASO Isis-414930 at 6.25, 12.5 and 25 mg/kg/week for about ten-week. Control ASO was administered at 25 mg/kg/week. Necropsy was performed 18-hr after the last dose of ASO. Liver, kidney and epididymal fat was dissected and extracted for RNA. The RNA was used to prepare cDNA by reverse transcription. Real-time quantitative RT-PCR (TaqMan) analysis demonstrated 81% and 63% reduction of CB1R mRNA in epididymal fat and kidney, respectively, in the 25 mg/kg/week group (Table 1). 12.5 mg/kg/week CB1R ASO treatment also significantly decreased CB1R mRNA in the epididymal fat (57% reduction), but not in the kidney. We detected very low level of CB1R mRNA in the liver and could not obtain any meaningful comparison between vehicle and CB1R ASO treated groups (data not shown). We also determined CB2R mRNA levels in epididymal fat and kidney and there were no significant changes (Table 1), confirming specificity of CB1R ASO.

**Table 1:** Relative CB1R and CB2R mRNA level after 10-week ASO Treatment.

Treatment	CB1R		CB2R	
	Epididymal fat	Kidney	Epididymal fat	Kidney
DIO Veh	1.16±0.25	1.08±0.18	0.91±0.10	1.03±0.10
CB1R ASO 25 mg/kg/wk	0.19±0.11*	0.37±0.02*	1.24±0.11	1.34±0.10
CB1R ASO 12.5 mg/kg/wk	0.43±0.16*	0.72±0.09	0.99±0.12	1.31±0.13
CB1R ASO 6.25 mg/kg/wk	0.88±0.29	0.66±0.04	0.84±0.10	1.32±0.10
Control ASO 25 mg/kg/wk	0.75±0.23	0.94±0.16	1.21±0.10	1.27±0.11

Values shown are fold change from DIO veh group for the same gene and tissue. Student t-test, compared to DIO Veh, *p<0.05.

### CB1R ASO Treatment Dose-dependently Reduced Body Weight Gain

The effects of CB1R ASO on body weight and food intake were observed after about 4-week treatment in a dose-dependent manner (Table 2). For veh-treated group, about 10% body gain was obtained during the study, while CB1R ASO 25 mg/kg/week group lost 2–3% of the starting body weight. The decreased body weight gain occurred concurrently with food intake decrease (Table 3). For instance, during week 4, food intake of CB1R ASO 25 mg/kg/week group was 20.0±0.6 g, compared to 22.3±0.6 g of veh-treated group, p<0.05. The extent of food intake reduction maintained to the end of the study. Neither body weight gain nor food intake was affected in control ASO treated group. Body fat mass was also evaluated on day 1 and the end of the study. CB1R ASO treatment produced a dose-dependent fat mass reduction with only 25 mg/kg/week group achieving statistic significance (Table 4).

**Table 2:** A. Body weight during the treatment.

A	BW (g)								
Treatment	Day 1	Day 12	Day 19	Day 26	Day 33	Day 40	Day 47	Day 54	Day 61
Lean Veh	35.8±0.3*	35.2±0.4*	35.5±0.4*	36.1±0.4*	36.0±0.4*	36.0±0.5*	35.5±0.4*	37.4±0.3*	36.3±0.4*
DIO Veh	46.9±1.0	46.8±1.0	46.6±1.6	48.0±1.1	47.6±1.1	49.9±1.1	49.9±0.9	52.0±0.8	51.2±0.9
CB1 ASO 25mg/kg/wk	47.3±0.9	47.5±0.9	47.6±0.9	47.1±1.0	46.5±1.0	46.6±0.8*	45.9±1.0*	47.3±0.9*	46.1±1.0*
CB1 ASO 12.5mg/kg/wk	47.9±1.2	47.9±1.3	48.0±1.3	48.2±1.4	48.0±1.5	48.2±1.7	48.3±1.6	50.4±1.5	49.4±1.4
CB1 ASO 6.25mg/kg/wk	47.6±1.1	47.4±1.3	48.6±1.1	48.6±1.1	48.9±1.2	49.2±1.2	49.2±1.1	50.9±1.2	49.1±1.3
Control ASO 25mg/kg/wk	47.3±1.0	47.3±1.0	47.8±1.0	48.4±1.1	48.8±1.2	49.3±1.2	49.9±1.2	51.9±1.2	50.5±1.3
B	BW (% of starting)								
Treatment	Day 1	Day 12	Day 19	Day 26	Day 33	Day 40	Day 47	Day 54	Day 61
DIO Veh	100±2.2	99.9±2.0	99.4±3.5	102.4±2.4	101.4±2.4	106.5±2.2	106.5±1.8	111.0±1.8	109.2±1.9
CB1 ASO 25mg/kg/wk	100±1.8	100.4±1.9	100.7±1.9	99.5±2.1 *	98.3±2.0*	98.5±1.7*	97.0±2.1 *	100.1±2.0*	97.6±2.0*
CB1 ASO 12.5mg/kg/wk	100±2.5	100±2.7	100.1±2.7	100.6±2.9	100.0±3.1	100.5±3.5	100.7±3.4	105.0±±3.2	102.9±2.9
CB1 ASO 6.25mg/kg/wk	100±2.4	99.5±2.8	100.2±2.2	102.1±2.4	102.6±2.5	103.4±2.4	103.3±2.4	106.9±2.4	103.3±2.8
Control ASO 25mg/kg/wk	100±2.0	100±2.1	101.0±2.1	102.3±2.3	103.2±2.5	104.3±2.5	105.4±2.6	109.8±2.5	106.8±2.7

B. Body weight change (% of the beginning body weight) during the treatment. One way ANOVA Dunnett’s multiple test compared to DIO Veh, *p<0.05.

**Table 3:** Food intake during the study.

	Weekly FI (g, mean ± se)							
Treatment	Wk 1&2	Wk 3	Wk 4	Wk 5	Wk 6	Wk 7	Wk 8	Wk 9
DIO Veh	23.7±0.5	23.4±0.6	22.3±0.6	22.9±0.5	22.6±0.3	25.1±0.7	22.3±0.4	22.0±0.5
CB1 ASO25 mg/kg/wk	22.7±0.6	21.1±0.6	20.0±0.6*	20.5±0.6	19.7±0.5*	22.3±0.6*	19.5±0.4*	19.4±0.4*
CB1 ASO12.5 mg/kg/wk	23.6±0.6	22.7±0.6	22.2±0.6	21.7±0.9	21.5±1.0	24.3±0.7	21.7±0.7	21.1±0.6
CB1 ASO6.25 mg/kg/wk	24.6±0.6	21.9±1.1	22.2±0.5	22.6±0.7	22.3±0.4	24.4±0.6	21.0±0.5	21.5±0.4
Control ASO25 mg/kg/wk	24.2±0.6	23.4±0.5	22.8±0.5	23.4±0.6	22.1±0.5	26.8±0.7	22.7±0.6	21.9±0.6

One way ANOVA Dunnett’s multiple test compared to DIO Veh, *p<0.05.

**Table 4:** Fat mass change before and after 10-week treatment.

Treatment	Fat (Day 1, % of total body mass,mean ± se)	Fat (Week 10, % of total body mass,mean ± se)	Fat Mass % change(mean ± se)
Lean Veh	19.3±0.6*	20.6±0.5*	1.4±1.0
DIO Veh	37.7±0.6	38.6±0.3	0.2±0.6
CB1R ASO 25 mg/kg/wk	38.8±0.6	34.0±0.7*	−4.5±0.8*
CB1R ASO 12.5 mg/kg/wk	39.3±0.5	37.5±0.5	−1.8±0.3
CB1R ASO 6.25 mg/kg/wk	39.4±0.8	38. 5±0.5	−1.0±1.1
Control ASO 25 mg/kg/wk	39.9±0.6	40.2±0.5	0.3±0.7

One way ANOVA Dunnett’s multiple test compared to DIO Veh, *p<0.05.

### CB1R ASO Treatment Improved Insulin Sensitivity and Glucose Homeostasis and Ameliorated Liver Steatosis

**Figure 1:**
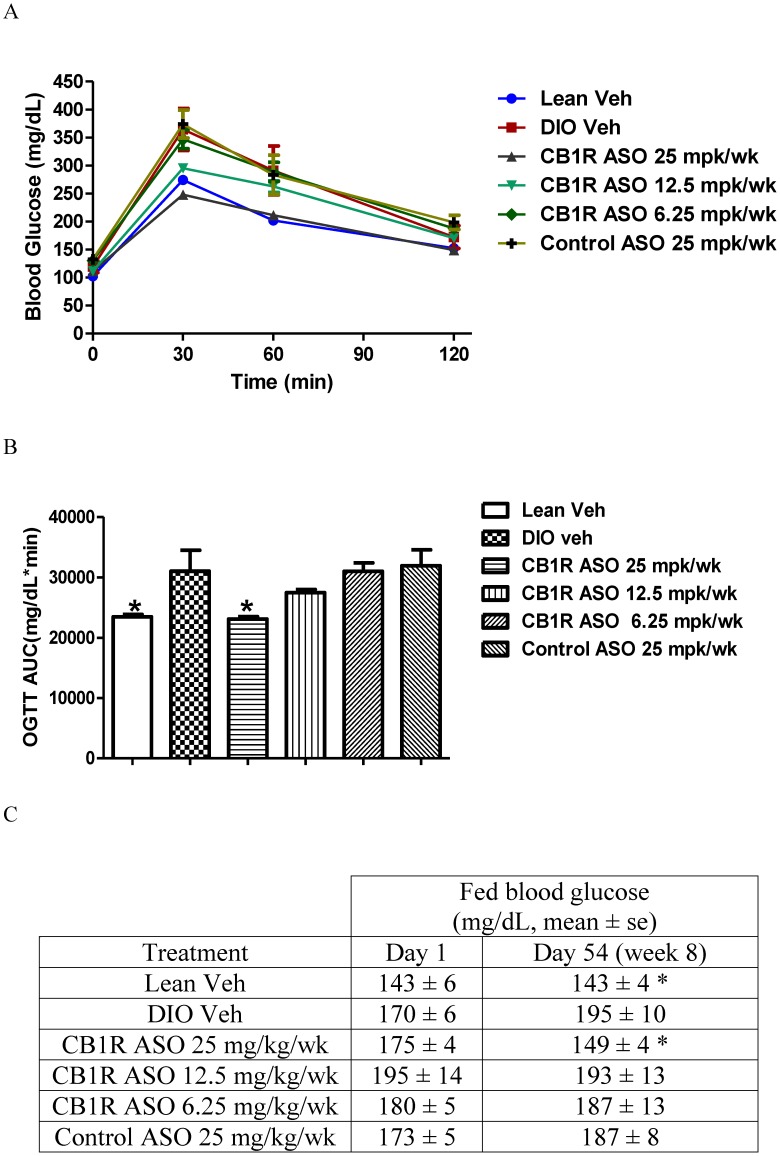
Oral glucose tolerance test (A and B). On Day 64, overnight fasted mice were challenged with oral glucose dosing at 2 g/kg. Blood glucose was measured at 0, 30, 60 and 120 minute after glucose dosing. Glucose area under curve was also calculated. C. Fed blood glucose was measured on day 1 and day 54. Student t-test vs the vehicle group, *, P<0.05.

Ad lib fed blood glucose was measured on day 54 (Figure 1). CB1R ASO treatment at 25 mg/kg/week reduced blood glucose from 195±10 mg/dL (veh-treated) to 149±4 mg/dL (p<0.05), while age-matched lean control group was at 143±4 mg/dL. Control ASO at 25 mg/kg/week and CB1R ASO at 12.5 and 6.25 mg/kg/week did not affect ad lib fed glucose level. Oral glucose tolerance was performed on day 64 (Figure 1). CB1R ASO 25 mg/kg/week treatment significantly improved glucose excursion to the level of age-matched lean control group (16±1% glucose AUC reduction for 25 mg/kg/week group from DIO veh group, p<0.05; lean control, 15±1% reduction from DIO veh, p<0.05). CB1R ASO 12.5 mg/kg/week administration tended to decrease glucose AUC, however to a lesser extent, 12±1% reduction from DIO veh group. Consistent with findings from OGTT, insulin level measured at the end of the study (week 10) clearly indicated the improved insulin sensitivity upon CB1R ASO treatment (Table 5). After CB1R ASO 25 mg/kg/week treatment, insulin level was normalized from 2.92±0.46 ng/mL to 0.69±0.09 ng/mL, a level very close to that of lean mice (0.51±0.08 ng/mL, despite the fact that body weight of CB1R ASO 25 mg/kg/week group was significantly higher than that of lean control group (46.1±1.0 g vs lean 36.3±0.4 g). Therefore, CB1R ASO treatment produced body-weight independent improvement in insulin sensitivity and glucose homeostasis. Liver TG was also determined at the end of the study (Table 6). CB1R ASO treatment led to dose-dependent reduction of liver TG content which may have in turn improved, at least in part, insulin sensitivity and glucose homeostasis.

**Table 5:** Plasma insulin, adiponectin, leptin, TG and cholesterol levels at the end of treatment.

Treatment	Insulin (ng/mL)	AdipQ (µg/mL)	Leptin (ng/mL)	TG (mg/dL)	Total cholesterol (mg/dL)
Lean	0.51±0.08*	24.0±1.3	7.21±0.51*	273±15*	92±2*
DIO Veh	2.92±0.46	21.2±0.9	32.47±1.20	178±9	158±4
CB1 ASO 25 mg/kg/wk	0.69±0.09*	25.9±1.1*	21.95±1.88*	189±18	111±4*
CB1 ASO 12.5 mg/kg/wk	1.73±0.35	23.9±1.1	30.73±1.34	196±13	141±5*
CB1 ASO 6.25 mg/kg/wk	2.04±0.38	21.4±1.0	30.93±1.73	193±21	147±6
Control ASO 25 mg/kg/wk	2.13±0.45	25.5±0.4*	32.80±1.11	195±16	170±4

Values shown are mean ± se. One way ANOVA Dunnett’s multiple test compared to DIO Veh, *p<0.05.

**Table 6:** Liver, spleen and epididymal fat weights, liver TG and plasma total PAI-1 at the end of treatment.

Treatment	Liver weight (g)	Liver TG (mg/g tissue)	Epididymal fat (g)	Spleen weight (g)	Plasma total PAI-1(pg/mL)
Lean Veh	1.82±0.05*	15±1*	1.17±0.07*	0.080±0.007	837±93*
DIO Veh	2.39±0.16	87±5	2.17±0.21	0.087±0.004	1722±244
CB1R ASO25 mg/kg/wk	1.97±0.09*	40±6*	1.99±0.16	0.138±0.009*	2765±327*
CB1R ASO12.5 mg/kg/wk	2.26±0.12	71±8	1.94±0.22	0.121±0.024	2145±278
CB1R ASO6.25 mg/kg/wk	2.21±0.10	81±12	2.08±0.15	0.093±0.003	1357±96
Control ASO25 mg/kg/wk	2.06±0.11	69±4	2.18±0.13	0.076±0.004	1180±83

Values shown are mean ± se. One way ANOVA Dunnett’s multiple test compared to DIO Veh, *p<0.05.

### Effects of CB1R ASO Treatment on Plasma Adiponectin, Leptin, TG, Total Cholesterol, Total PAI-1 and Tissue Weights

Consistent with reduced adiposity demonstrated by fat mass measurement with NMR, plasma leptin level was decreased upon CB1R ASO 25 mg/kg/week treatment, indicating reduced hyperleptinemia induced by high fat diet (Table 5). Total cholesterol level was decreased as well. However, plasma TG level was not changed. Plasma total adiponectin level was slightly increased upon CB1R ASO 25 mg/kg/week treatment (25.9±1.1 µg/mL vs. DIO veh group, 21.2±0.9 µg/mL, p<0.05). However, plasma adiponectin level of control ASO 25 mg/kg/week treatment group was similar to that of CB1R ASO 25 mg/kg/week group. In addition, CB1R ASO 25 mg/kg/week treatment led to reduction of liver weight which is consistent with improved liver steatosis (Table 6). Epididymal fat weight was not significantly altered upon CB1R ASO treatment (Table 6). Spleen weight was increased upon CB1R ASO 25 mg/kg/week treatment (Table 6). This phenomenon is often observed upon ASO treatment and related to induction of pro-inflammatory state. Therefore, we also measured plasma TNFα, IL-6 and total plasminogen activator inhibitor 1 (PAI-1) levels. There were no significant changes for plasma TNFα and IL-6 under the circumstances that levels of both cytokines in DIO veh group were at the very low end of the standard curves (data not shown). However, plasma total PAI-1 level was elevated which seemingly correlated to the spleen weight. Plasma PAI-1 level tends to be increased under inflammatory conditions and its production was shown to be increased by TNF-α [32], [33].

### Reduced mRNA Levels for Genes Involved in Gluconeogenesis, De Novo Lipogenesis and Fatty Acid Transport

In order to further understand the mechanisms underlying improved glucose homeostasis and reduced hepatic steatosis, we examined the mRNA levels of several genes that play key roles in gluconeogenesis and lipogenesis in the liver and epididymal fat tissue. As shown in Table 7, for the master regulator of lipid synthesis SREBP-1, its mRNA level was significantly reduced in both liver and epididymal fat upon CB1R ASO 25 mg/kg/week treatment. mRNA level of glucose 6-phosphatase catalytic unit (G6PC) was also decreased in the liver. In addition, fatty acid translocase/CD36 was also reduced in liver, consistent with improved hepatic steatosis.

**Table 7:** mRNA levels of several genes in epididymal fat and liver at the end of the study.

Tissue	Gene	DIO Veh	CB1R ASO25 mg/kg/week	CB1R ASO12.5 mg/kg/week	CB1R ASO6.25 mg/kg/week	Control ASO25 mg/kg/week
Liver	SREBP-1	1.02±0.06	0.74±0.06*	0.73±0.08*	0.90±0.05	0.95±0.11
Liver	G6PC	1.15±0.23	0.43±0.09*	0.68±0.13	0.91±0.13	0.99±0.13
Liver	CD36	1.20±0.30	0.20±0.02*	0.44±0.10*	0.86±0.13	0.53±0.13
Epididymal fat	SREBP-1	1.03±0.10	0.57±0.11*	0.73±0.13	0.83±0.15	0.75±0.13

Values shown are mean ± se. Student t- test, compared to DIO Veh, *p<0.05.

### Clinical Chemistry Parameters Unchanged upon CB1 ASO Treatment

Plasma levels of creatinine, BUN, lipase and liver enzymes (ALT and AST) were measured at the end of the 10-week study to collectively monitor any treatment-induced adverse renal, pancreatic and hepatic effects. As shown in Table 8, there was no significant difference between lean veh and DIO veh groups with regard to plasma creatinine, BUN and lipase levels. ASO treatment did not lead to any changes in these parameters, either. For liver enzymes, both ALT and AST levels were elevated in DIO veh group compared to that of lean veh group, which is a general phenomenon related to high fat diet-induced steatosis. ALT level appeared to be reduced upon CB1 ASO 25 mg/kg/wk treatment. However, it’s unclear why the control ASO group exhibited reduced plasma ALT and AST levels. In summary, the overall well-being of the mice was not significantly affected by 10-week CB1R or control ASO treatment.

**Table 8:** Plasma creatinine, BUN, lipase, ALT and AST levels at the end of treatment.

Treatment	Creatinine (mg/dL)	BUN (mg/dL)	Lipase (U/L)	ALT (U/L)	AST (U/L)
Lean Veh	0.32±0.01	18.5±0.7	76±12	54±14*	64±6*
DIO Veh	0.35±0.01	16.1±0.9	86±2	125±11	106±12
CB1 ASO 25 mg/kg/wk	0.33±0.00	14.9±0.9	70±4	80±6*	103±12
CB1 ASO 12.5 mg/kg/wk	0.36±0.02	17.5±1.5	89±5	122±10	138±32
CB1 ASO 6.25 mg/kg/wk	0.33±0.01	15.9±0.3	97±10	103±15	85±9
Control ASO 25 mg/kg/wk	0.47±0.13	17.1±0.3	90±2	73±7*	71±5*

Values shown are mean ± se. One way ANOVA Dunnett’s multiple test compared to DIO Veh, *p<0.05.

## Discussion

Since anti-sense oligonucleotides do not cross the blood brain barrier, we utilized this approach to investigate the metabolic consequencies of inhibiting CB1R function in peripheral tissues. High-fat diet has been shown to increase plasma and/or tissue endocannaibinoid levels which over-stimulates CB1R in the peripheral tissues [15], [16], [34], leading to dysregulation of many metabolic pathways. Therefore, we performed the 10-week study with a specific CB1R ASO in diet-induced obese AKR/J mice. We demonstrated that CB1R ASO treatment improved glycemic control and reversed hepatic steatosis. CB1R was found to express at highest level in the CNS [30]. Although CB1R expression at the peripheral tissues was much lower than that of CNS, its functional relevance was well demonstrated (for review, see [1], [12], [23], [31] ). In the present study, the rank order of CB1R mRNA level in diet-induced obese AKR/J mice was adipose>kidney>liver. In both pre-clinical animal models and clinical trials, inhibition of CB1R by antagonists such as rimonabant led to initial weight loss caused by transient reduction in food consumption (for review, see [11], [12]). Although tolerance to anorectic effect of brain penetrant CB1R antagonists quickly developed, body weight loss was sustained [35], [36]. Therefore, the long-lasting metabolic effects of CB1R antagonists such as rimonabant cannot be attributed entirely to the reduced caloric intake and weight loss. Instead, an increase in energy expenditure and improvement in lipid metabolism and glucose homeostasis may have occurred in peripheral tissues. Here we observed approximately 10% body weight gain reduction after 4–5 week treatment with CB1R ASO 25 mg/kg/week which sustained to the end of the study. In parallel, there was a small reduction in food intake. Beyond the orexigenic effects CB1R plays in the hypothalamus, CB1R also co-expresses with satiety hormone cholecystokinin in vagal afferent neurons and may influence food intake by mediating satiety signaling in the gut [20]. Similar to findings in DIO mice treated with CB1R antagonists such as rimonabant, body weight gain reduction upon ASO treatment was correlated with fat mass decrease which may be derived from increased lipolysis and fatty acid oxidation [35], [36]. The white adipose tissue has been commonly recognized to be an endocrine organ which plays a central role in many metabolic pathways via secretion of various adipokines including adiponectin and leptin. The physiological roles of CB1R in adipocytes and adipose tissue have been extensively explored and well established. In primary adipocytes, blockade of CB1R led to enhanced secretion of adiponectin and mitochondria biogenesis as well as decrease of lipogenic gene expression (e.g., SCD1 and FAS) [23], [24], [37]. In animal models of obesity and diabetes [38], [39], [40] as well as in obese subjects [41], [42], treatment with CB1R antagonists such as rimonabant has led to increased plasma adiponectin level, in particular the high molecular weight adiponectin. Adiponectin increases glucose uptake and fatty acid oxidation in skeletal muscle, reduces hepatic glucose production and improves overall insulin sensitivity [43]. Here in the present study, we did observe an apparent dose-dependent total adiponectin increase upon CB1R ASO treatment, although this finding was complicated by the increased adiponection level from the ASO control group. It’s possible that the HMW adiponectin level profile was more reflective of the metabolic benefits upon CB1R ASO treatment since this form of adiponectin has been shown to possess the most potent insulin-sensitizing activity [40], [44]. High-fat diet is also known to trigger a peripheral leptin resistance and hyperleptinemia [45], [46]. Leptin was shown to stimulate fatty acid oxidation by activating AMP-activate protein kinase in skeletal muscle [47]. Here in this study, high-fat diet significantly increased plasma leptin level and CB1R ASO treatment normalized that, indicating amelioration of leptin resistance (Table 5). Our findings are consistent with reported reduction of plasma leptin level upon administration of rimonabant and a peripherally restricted CB1R antagonist AM6545 in DIO C57Bl/6 [48]. An alternative mechanism for CB1R ASO action in white adipose tissue (WAT) is inhibition of CB1R in the sympathetic nerve terminals. CB1R was shown to express at the peripheral sympathetic nerve terminals and inhibit norepinepherine release upon activation [49]. It’s commonly known that norepinepherine activates hormone sensitive lipase and consequently stimulates lipolysis in the adipose tissue via adrenergic receptors [50]. Furthermore, a recent report by Quarta et al demonstrated the key role of sympathetic nervous system (SNS) in rimonabant-mediated increase of lipid utilization and energy expenditure by using a conditional knock-out of CB1R in the forebrain and sympathetic neurons [51]. The authors did recognize that relative contribution from CNS versus SNS endoconnabinoid signaling in the control of peripheral energy balance remained to be understood, possibly by specific knock-out of CB1R in the sympathetic ganglion [51]. In addition to WAT, brown adipose tissue (BAT) was also shown to be under the SNS control and account for rimonabant-stimulated thermogenesis in BAT and possibly in part, the body weight loss [36], [51], [52]. Here in the present study, we showed that fat mass was significantly reduced upon peripheral blockade upon CB1R ASO treatment, implicating enhanced lipolysis and decreased lipogenesis. This was supported by reduced SREBP-1 mRNA level in epididymal fat (Table 7). Similarly, a peripherally restricted CB1R antagonist AM6545 in DIO C57Bl/6 decreased lipogenic gene expression (e.g., SCD1 and FAS) in both subcutaneous and visceral fats [51]. CB1R was also found in human adipose tissue and the endocannabinoid system was up-regulated in obese subjects [17], [18]. Therefore, findings in preclinical animal models may be translated in humans.

The liver plays a pivotal role in glucose homeostasis and lipid metabolism. Liver expresses a low level of CB1R [6], [53], [54], [55]. Nonetheless, activation of CB1 in mice increases hepatic gene expression of the lipogenic transcription factor SREBP-1 and the lipogenic genes acetyl-CoA carboxylase-1 (ACC1) and fatty acid synthase (FAS) [6]. Furthermore, hepatocyte-selective genetic knockout of CB1R in mice led to reduced steatosis, hyperglycemia and insulin and leptin resistance on high-fat diet in the context of similar adiposity when compared to wild type mice on high-fat diet [6], [56]. Other cells in the liver such as stellate cells may also affect lipid metabolism by exerting paracrine effects on hepatocyte CB1R [57]. Here in this report, after CB1R ASO treatment, liver TG was reduced (Table 6), indicating ameliorated hepatic steatosis. Our RT-PCR analysis of liver samples suggested again the very low level of CB1R expression in these mice even after HFD feeding (data not shown). It may be possible that the cross-talk of adipose tissue and liver via altered secretion of adipokines and inflammatory cytokines plays a more important role than modulation of CB1R per se in the liver [39], [40], [58], [59]. CB1R was also found in skeletal muscle and pancreas. Rimonbant treatment led to increased glucose uptake in skeletal muscle [60]. The roles of CB1R play in the pancreas remain to be fully understood. Activation of CB1R in mouse pancreatic islets led to inhibition of insulin secretion [61], while rimonabant treatment in Zucker fa/fa rats increased glucose-dependent insulin secretion index, indicating a protective role of rimonabant in β-cell function [25], [26], [61]. In isolated human islets, CB1R was found to express more densely in the alpha cells than in the beta cells [8]. CB1 stimulation enhanced insulin and glucagon secretion in human islets [8]. The roles CB1R play in the pancreas remains to be fully understood. Since anti-sense oligonucleotides generally don’t distribute to skeletal muscle and pancreas well, the improved metabolic profile here may not be attributed to reduction of CB1R expression in these tissues. Glucose homeostasis and insulin sensitivity was improved upon CB1R ASO treatment as demonstrated by oral glucose tolerance test and reduced plasma insulin level (Figure 1 and Table 5). It’s generally realized that ASO chronic treatment in animals tended to induce pro-inflammatory status. Here we observed the dose-dependent increase of spleen weight and total plasma PAI-1 level upon CB1R ASO treatment, although both spleen weight and PAI-1 level of control ASO group was similar to that of DIO vehicle-group. This seemingly up-regulated pro-inflammatory state may worsen insulin resistance in diet-induced obesity in this study. Nonetheless, overall insulin resistance and glucose homeostasis was improved, strongly suggesting that reduction of CB1R expression exerted a more predominant role in the peripheral tissues. This result is consistent with findings in diet-induced obese mice when treated with CB1 antagonists [34], [39], [59]. Kidney also contributes substantially to total-body glucose homeostasis by regulating gluconeogenesis, filtering and reabsorbing glucose [62], [63]. Here we showed CB1R ASO treatment led to significant reduction of CB1 receptor in the kidney. Whether or not CB1R plays a role in renal glucose release and the consequences of CB1R blockade remain to be explored.

We do recognize some important limitations of this study here. Although ASOs are well known not to penetrate blood brain barrier, compensatory mechanism may lead to changes of the endocannabinoid system in the central nervous system (CNS) after prolonged reduction of CB1R in peripheral tissues. Therefore we cannot totally rule out the contribution of the CNS to the observed metabolic benefits here. Based on our and others’ experience, steady state mRNA level after chronic ASO treatment generally correlates well with protein level of the target gene. Due to this belief, we did not perform analysis of CB1R protein level in target tissues in this study. In retrospect, confirmation of CB1R protein level could have made a more compelling case. The results here should be interpretated with caution.

Finally, we also recognize that the endocannabinoid system also contains CB2R. Although CB2R is less understood than CB1R with regard to its roles in metabolic disorders, evidvence has emerged suggesting the potential involvement of CB2R in glucose homeostasis, hepatic steatosis and obesity-associated inflammation [64], [65], [66], [67], [68]. In this study, we also measured the mRNA level of CB2R in epididymal fat, there was no change upon CB1R or control ASO treatment.Nonetheless, since plasma or tissue endocannabinoids levels were not determined in this study, we cannot completely rule out the possible contribution of CB2R to the observed metabolic benefits.

In summary, we demonstrated here that selective knockdown of the peripheral CB1R using an ASO approach led to improved insulin sensitivity and glucose homeostasis in DIO AKR/J mice. Liver steatosis associated with DIO was also ameliorated upon CB1R ASO treatment. These findings support the notion that inhibition of CB1R in peripheral tissues may exert significant beneficial metabolic benefits and may represent a novel therapeutic approach for treating diabetes and related metabolic syndrome.
